# Viruses in Extreme Environments, Current Overview, and Biotechnological Potential

**DOI:** 10.3390/v13010081

**Published:** 2021-01-08

**Authors:** Jose F. Gil, Victoria Mesa, Natalia Estrada-Ortiz, Mauricio Lopez-Obando, Andrés Gómez, Jersson Plácido

**Affiliations:** 1 VEDAS Corporación de Investigación e Innovación (VEDAS CII), Medellín 050024, Colombia; victoria.mesa@vedascii.org (V.M.); natalia.estrada@vedascii.org (N.E.-O.); mauricio.obando@vedascii.org (M.L.-O.); 2 Department of Animal Science, Food Science and Nutrition, University of Minnesota, St. Paul, MN 55108-6118, USA; gomeza@umn.edu; 3 Institute of Life Science, Medical School, Swansea University, Swansea SA2 8PP, UK

**Keywords:** extremophile viruses, virosphere, eukaryotic viruses, archaeal viruses, bacterial viruses

## Abstract

Virus research has advanced significantly since the discovery of the tobacco mosaic virus (TMV), the characterization of its infection mechanisms and the factors that determine their pathogenicity. However, most viral research has focused on pathogenic viruses to humans, animals and plants, which represent only a small fraction in the virosphere. As a result, the role of most viral genes, and the mechanisms of coevolution between mutualistic viruses, their host and their environment, beyond pathogenicity, remain poorly understood. This review focuses on general aspects of viruses that interact with extremophile organisms, characteristics and examples of mechanisms of adaptation. Finally, this review provides an overview on how knowledge of extremophile viruses sheds light on the application of new tools of relevant use in modern molecular biology, discussing their value in a biotechnological context.

## 1. Introduction

Extremophile organisms are found in hostile environments; places where life was thought not to be possible. Facultative-adapted organisms, known as extremotolerant, can be found from toxic waste, acid and alkaline lakes, to high pressure, ice or extremely hot environments. Hence, organisms that require these extreme environments to survive are classified (or known) as extremophilic [1].

Depending on the environment that these organisms are isolated from, they can be classified as thermophiles (and hyperthermophiles: from very high temperatures), psychrophiles (from low temperatures), acidophiles and alkaliphiles (from acid or alkaline environments), barophiles (from high pressure environments) and halophiles (from saline environments) [1,2] (See [2] for details). Extremophiles, either extremotolerant or extremophilic, are highly diverse, including species in the three domains of life: bacteria, archaea and eukaryote. Recently, it has been possible to extend their sampling coverage; partially due to climate change, which has made possible to find extremophiles in places where it was not possible to reach out before. Moreover, due to advances in sampling and sequencing technologies (e.g., high-throughput sequencing), it has been possible to access more information on the taxonomy and function of these organisms [3].

The discovery of virus infecting these organisms resulted from investigating microbial communities in extreme environments, indicating that all living organisms on earth are hosts of viruses and that viruses are the most abundant biological entities on the planet [4]. Moreover, it has been established that extremophiles act as hosts for viral replication and that viruses are responsible for the balance of population dynamics in extreme environments [5]. In this regard, several studies have analyzed prokaryotic (Bacteria and Archaea) and Eukaryotic abundance and diversity along different extreme environments [6,7,8]. In contrast, little is known about their viral co-symbionts and their ecological roles across niches within harsh life conditions around the world [9]. Furthermore, numerous ecological studies have revealed that prokaryotic viruses predominate across different environments, including extreme niches, and outnumbering their hosts by at least an order of magnitude [10,11]. Thus, documenting viral diversity in extremophile hosts, from molecular and taxonomic perspectives, provides vital information about how viruses shaped life on earth and about the viral molecular mechanisms involved in this process, which could constitute a source of novel molecular tools for biotechnological applications. This review focuses on what is known about viruses infecting extremophile organisms, and brings particular examples on how research on extremophile viruses sheds light on their potential use as sources for biotechnological tools.

## 2. Viruses of Extremophile Archaea

Archaea are organisms that share several biological characteristics with both bacteria and eukaryotes, and such similarities vary depending on taxonomic classification (Reviewed in [12]). Archaea were considered only to thrive in extreme environments; however, they have been found in several mesophilic ecosystems, where they flourish thanks to the acquisition of adaptive genes from bacteria [13,14,15,16]. One of the most interesting findings was the identification of archaea on the human body. Among them, methanogens found in the colon represent an important community with potentially defining roles in digestive function, along with those colonizing the subgingival plaque, and skin [17]. To add-on their captivating lifestyles, research on extremophilic archaea is receiving increasing attention due to the viruses that have been found associated with their populations. These observations suggest that viruses infecting archaea might represent the most diverse and abundant biological entities on earth [18].

Viruses infecting archaea have an important role in influencing global biogeochemical cycles; they are responsible for the production of approximately 0.3 to 0.5 gigatonnes of carbon per year, only by lysis of archaeal cells in the top 50 cm in the surface deep-sea sediments [19]. Despite this important role, little is known about their genomic characteristics. Globally, ~75–90% of genes in archaeal viruses (depending on the taxa) lack significant sequence match or homology in public databases, making their functional annotation difficult and limiting knowledge on their biology and specific functions in extreme niches [12,20]. Table 1 summarizes relevant examples of viruses isolated from extremophile archaea and their main taxonomic features [12,21,22].

Archaeal viruses classified so far are grouped within DNA viruses, both having dsDNA and ssDNA genomes. Viruses within the dsDNA group have been divided in 13 families: *Ampullaviridae* (bottle-shaped), *Bicaudaviridae* (spindle-shaped), *Clavaviridae* (club, stick), *Fuselloviridae* (spindle-shaped), *Guttaviridae* (droplet-shaped), *Globuloviridae* (round-shaped), *Lipothrixviridae* (fat hair-shaped), *Myoviridae* (muscle, referring to the contractile tail), *Portogloboviridae* (carrying ball), *Rudiviridae* (small rod), *Sphaerolipoviridae* (fat sphere), *Tristromaviridae* (three layer) and *Turriviridae* (turret-like appendages expanding from the capsid); while ssDNA group contains only two families *Pleolipoviridae* (many lipid) and *Spiraviridae* (coil-shaped) [12,32]. *Myoviridae* is the only family, to date, that infects both bacteria and archaea [32].

The biggest challenge faced when attempting to characterize archaeal viruses is the difficulty with isolation and culturing their hosts. Due to the high diversity of virion morphotypes and genome content of archaeal viruses, it is believed that we are just starting to grasp the real diversity of this group of viruses. This diversity has derived in the creation of taxonomic families that better explain and fit archaeal viruses’ characteristics [33].

A better understanding of the interactions between archaea and their viruses, and the impact of viral co-symbionts on community ecology requires studying the environments they inhabit, along with the detailed taxonomy, morphology and functional genomics of the isolated viruses. This also includes the study of viral mechanisms involved in host adaptation to extreme environments, [21,34,35,36]. For example, in hyperthermophilic environments, viruses classified within the family *Fuselloviridae* infect the thermoacidophilic genus *Sulfolobus* [37,38]. Organisms within this genus have been used as a model to understand archaeal interactions with their viruses, including immune system responses [38]. *Sulfolobus* spindle-shaped virus 1 (SSV1) (dsDNA) is highly tolerant to mutations, being able to evade insertions and deletions in ~50% of its coding sequence, which is the same proportion of genes that are not essential for infectivity [39]. These non-essential ORFs (coding for <100 aa putative proteins) might be non-coding RNAs [39], which can act as regulatory molecules in the host [40]. Possibly, these genes, which are susceptible to modifications but that have no impact on the integrity of the virus and its infectivity, are or were required for adaptation to compete with other viruses or environmental conditions [39]. So far, these remain as hypotheses to be tested. In contrast, other members of the *Fuselloviridae* family, such as SSV10, encode a putative CRISPR associated Cas4-like ORF [38]. This protein is predicted to show high reliability and matches with several Cas4 and RecB (3′ to 5′ helicase, nuclease) protein structures found in several bacteria and archaea, as well as conserved motifs and active sites, among which 5′ to 3′ exonuclease activity is highlighted [38]. It is speculated that the protein produced from this ORF may have a counter activity against archaeal immune responses or, a multifunctional response against different stress responses (discussed in [38]).

Other mechanisms that mediate adaptation to extreme environments have been described such as a form of virion organization. For example, packing A-form DNA and disulfides in intracellular viral proteins. In the first one, almost half of the capsid protein is unstructured in solution, this unstructured region folds in the virion into a single extended α-helix which wraps around the DNA. The DNA is entirely in A-form, suggesting a mechanism for protecting DNA in the most adverse environments. The finding that the genomic dsDNA within the icosahedral capsid adopts an A-form has been reported in viruses as *Sulfolobus* polyhedral virus 1 (SPV1) and *Sulfolobus* SIRV2 [29,41]. In the second mechanism mentioned, intracellular disulfide bonds are present. These bonds are common in cellular proteins of *Sulfolobus* turreted icosahedral virus (STIV), in which a 93-residue protein has been reported and its characterization reveals a homodimeric winged-helix protein that is likely to function as a transcriptional regulator, suggesting the enhancing of the thermostability of the viral proteome [42].

## 3. Viruses of Extremophile Bacteria

Bacterial viruses (also known as bacteriophages or phages) are estimated to be the most abundant and diverse biological entities on the planet [43]; therefore, detectable in almost every biological niche, representing a vast source of biodiversity [44,45]. They can influence the abundance, diversity and evolution of bacterial communities; for instance, by supporting ecosystem homeostasis, as reported in marine ecosystems [43]. In these environments, viruses control prokaryotic and phytoplankton mortality, influencing microbial diversity and niche metabolic turnover [46]. For example, in geothermal environments, thermophilic phages also play significant roles in biogeochemistry, ecology and genetic exchange [47,48]. These roles are also common in archaeal communities, which highlights the importance of viruses for global ecology dynamics.

Bacteriophages are found as DNA or RNA with single- and double-stranded genomes [32]. The reported bacterial virus dsDNA families belong to the order Caudovirales (dsDNA) as *Myoviridae*, *Siphoviridae* and *Podoviridae*. In addition, viruses in the families *Tectiviridae*, *Corticoviridae*, *Plasmaviridae* and *Sphaerolipoviridae* have dsDNA genomes, but are not grouped within a taxonomical order. ssDNA viruses include families *Microviridae* and *Inoviridae*. Families *Cystoviridae* and *Picobirnaviridae* are grouped within dsRNA viruses, while family *Leviviridae* in ssRNA viruses [32]. Altogether viruses classified within these taxonomic families infect more than 140 bacterial genera and their classification depends greatly on their morphotypes and host genera. About 96% of phages are tailed, from which 61% correspond to *Siphoviridae*. The other types of phages (3.6%) are cubic, filamentous, or pleomorphic [10,45,49].

*Geobacillus kaustophilus*, *Bacillus stearothermophilus* and *Thermus* strains commonly harbor viruses within the families *Siphoviridae* and *Myoviridae* in thermophilic environments [48,50,51,52]. To date, little is known about bacterial population inhabiting hypersaline environments distributed globally in the form of salt lakes and salt ponds, and even less is known about their viral co-symbionts [53]. Ten viruses have been identified infecting halobacteria, particularly *Deleya halophile*, *Halomonas halophile*, *Pseudomonas* sp., *Salinivibrio costicolan* sp., *Salicola* sp., *Salisaeta* sp., *Salinivibrio* sp. In the case of cryophilic environments, the prevailing viral families are *Myoviridae* and *Siphoviridae*, infecting bacteria within the genera *Shewanella*, *Flavobacterium* and *Colwellia*. On the other hand, in acidic systems, the species *Acidithiobacillus caldus* has been associated with viruses from the *Myoviridae* family [54]. Additional relevant examples of viruses infecting bacteria are listed in Table 2.

In psychrophilic phage–host interactions, bacteriophage 9A, which infects the marine psychrophilic gamma-proteobacterium *Colwellia psychrerythraea* strain 34H (*Cp34H*) (temperatures between −12 and 8 °C), has been analyzed to investigate the potential role of 9A genes in host adaptations to cold temperatures. Colangelo-Lillis et al. [56] identified candidates for auxiliary metabolic genes (AMGs) involved in this adaptation, such as the homolog of a cold-active alkaline serine protease (*hp-122*, which is reported to enhance nutrient acquisition during phage replication), an extracellular enzyme of *Cp34H*, and several genes related to phosphate metabolism (e.g., nicotinamide mononucleotide adenylyltransferase, nicotinamide phosphoribosyl transferase). Other 9A genes may also be involved in facilitating interactions between phage and host; such as the genes 9A homolog (*hp-132*) of *Cp34H* histone deacetylase and 9A homolog (*hp-141*) of *Cp34H* DNA topoisomerase III. The *hp-132* gene is involved in high-affinity binding between histones and DNA and it might inhibit transcription of proteins involved in defense of the host. The *hp-141* is believed to be involved in adaptation to cold stress [56].

## 4. Viruses of Extremophile Eucaryotes

The abundance and diversity of eukaryotic extremophiles is significantly lower compared to what has been observed in extremophile bacteria and archaea; therefore, the diversity of viruses in eukaryotic extremophiles has been less documented. Bacteria, archaea and eukaryotes show domain specific viromes and mobilomes, with archaea and bacteria sharing numerous dsDNA virus families [61]. However, Eukaryotes host a greater diversity of RNA viruses, reverse transcribing elements and retroviruses than archaea and bacteria [61]. As more advances in genomics and in metabolomics techniques uncover eukaryotic diversity and function in extreme conditions (reviewed in [62]), more information about their viral co-symbionts will be revealed.

Deep sea and polar conditions are the main extreme environments in which viral infections have been reported, primarily in extremophile animals [63,64]. These studies have helped to predict epidemiological risks of mesophilic viruses. An example of viruses infecting eukaryotic extremophiles is seen in artic (tundra-boreal) environments between non-hematophagous insects (*Chaoborus* spp.) and phasmaviruses (*Bunyaviridae*) in artic environments; in what seems to be a long-term coevolutionary relationship between virus and host [65]. For instance, the Kigluaik phantom virus (KIGV) infects polar phantom midges and has been transmitted vertically in *Chaoborus trivittatus* (North American populations) for thousands of years. The high prevalence of the virus in the insect population is evidence that this coevolutionary relationship leads to endosymbiosis [65]. Table 3 describes the characteristics of viruses found in extremophile animals.

Regarding microorganisms, the greatest number of viruses of eukaryotes has been described in deep-sea phytoplankton [69] and polar environments [70]; nevertheless, geothermal locations (soil and lakes) are also a source of extremophile eukaryotic microorganisms and their viruses [71]. Table 4 details some of the characteristics associated with viruses found infecting extremophile eukaryotic microorganisms.

In polar environments and deep-sea, the principal source of viruses in eukaryotic microorganisms is associated with the viral shunt, an ecological mechanism that allows microbial recycling processes in the oceans [72]. *Micromonas pusilla* viruses (MpV) have been studied under different conditions, such as temperature, carbon dioxide (CO_2_), phosphorous limitation and iron limitation [73]. The MpV viral burst is affected by phosphorus and iron limiting conditions, these limited conditions prolong its latency period [73] and reduce its infectivity in about 70% [74]. In contrast, a steady low supply of soluble reactive phosphorus increases the viral burst [69].

With the advance of methodologies for massive parallel sequencing and metagenomics, it has become increasingly easier to study viromes of eukaryotic organisms in extreme environments [5]. This is the case of *Emiliania huxleyi* phytoplankton (coccolithophore), large dsDNA viruses (EhV, *Coccolithoviridae*), for which information about its genomic properties has been detailed [75]. The virus–host interaction (EhV—*E. huxleyi*) has provided important information to understand early cellular and virus evolution [76]. Compared with the temperate counterpart, polar EhV have different genomic features such as hypervariable region and the presence of two specific tRNAs, a phosphate permease, an endonuclease and a transposase [75,77]. A remarkable feature in the EhVs genome is the presence of genes involved in metabolism (such as AMG), a feature thought to be present only in the hosts [78]. EhV have the ability to downregulate host genes involved in de novo sphingolipid biosynthesis of the host, while the viral genes involved in the same pathway are upregulated (e.g., serine palmitoyltransferase). These observations indicate that the virus hijacks the host sphingolipid biosynthesis functions [70]. All these metabolic changes help the virus control mortality of *E. huxleyi*, while evading the phytoplankton defense mechanisms [70,78]. For example, production of signaling lipids microdomains help with recognition of the virion by the *E. huxleyi* cell membrane; nonetheless, this mechanism also simultaneously ensures that the structural properties of the virions are protected from the environment in the north Atlantic, due to the multihydroxylated ceramide backbone membranes produced by the host [70].

Extreme viruses in eukaryotic microorganisms have also been described in geothermal lakes and soils [71,79]. The three-way mutualistic symbiotic relationship among the fungus *Curvularia protuberata*, the mycovirus *Curvularia* thermal tolerance virus (CThTV) and panic grass (*Dichanthelium lanuginosum*) allows all three organisms to survive in geothermal soils at 65 °C; an environment they would not survive in individually [79,80]. In fact, the absence of viral infection on *C. protuberata* leads to lack of thermotolerance by the plant and the fungus [80]. Other plant hosts have gained thermotolerance after being mixed with infected *C. protuberata*, pointing to the conserved nature of the resistance mechanism conferred by viruses to the host [79]. Genes in *C. protuberata* activated by CThTV are associated with the production of osmoprotectants, such as glycine betaine, taurine and trehalose. Additionally, scytalone dehydratase, a key enzyme in the melanin production pathway, increased 10-fold in the infected strains, pointing to a potential role of melanin on the fungus thermotolerance [79].

In the case of plants, those living under polar and drought conditions are the main source of currently described extremophile viruses. In polar conditions, an example are two viral entities isolated from 700-year-old caribou feces. The ancient caribou feces associated virus (aCFV) and the ancient northwest territories cripavirus (aNCV) [81]. It has been suggested that those viruses could have originated from plant material consumed by the caribou from tundra environments. In fact, the authors successfully infected *N. benthamiana* with a cloned aCFV; the virus was able to replicate and spread systemically in the plant [81]. The sequences reported for this virus were significantly different from previously published viral sequences, indicating the unexplored nature of subarctic plant viruses (current and ancient) [81]. Table 5 summarizes the most relevant characteristics of these viruses.

## 5. Biotechnological Potential

The growing interest on the molecular dynamics of extremophiles and their viruses resides mainly on the premise that understanding these systems will improve understanding protein folding, stability, protein–protein interactions and even, the influence viruses have on host evolution [38].

**Figure 1:**
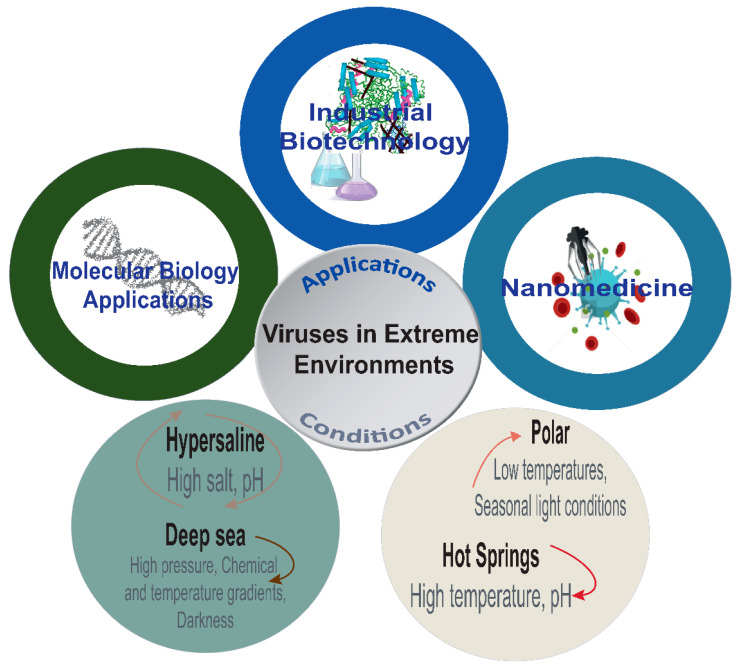
Main features of the extreme conditions and potential applications of extremophile viruses. Physical and chemical characteristics modeling the different environments and the potential biotechnological applications of viruses including molecular biology applications, nano-medicine and drug delivery and industrial biotechnology.

The number of viral genome sequencing projects reported to date, July 2020, in the National Center for Biotechnology Information (NCBI) Genome database indicated that 9735 viral genomes have been sequenced where 2929 were isolated from bacteria, 89 from archaea, 39 from eukaryotic algae, 272 from fungi, 40 from protozoa and 1823 from land plants [91,92]. However, the genomic potential of the viral genomes sequenced up to date, particularly, of extremophile viruses, for biotechnological applications remains a largely unexplored subject [5]. Extremophile organisms (bacteria and archaea) have shown to be a very rich source of thermostable enzymes with industrial applications, going from polymerase for molecular biology to lipases and proteases in detergents for processes that take place at extreme conditions, such as high or low temperatures and pH and the production of biopolymers [93,94]. Enzymes from extremophile viruses will have a critical role in complementing such processes and finding new niches (Figure 1).

### 5.1. Molecular Biology

Environmental sampling in tandem with recent advances in sequencing methodologies, meta-OMICS and isolation techniques have brought to light novel discoveries in virology, particularly in extremophile organisms, that were almost inaccessible in the past (e.g., endocytobiont pandoravirus [95]). The appeal of extremophile viruses relies on their particular ability to adapt to difficult environments and the molecular mechanisms that allow them to bypass their hosts’ defense strategies, hence, being successful at replicating and producing stable virions in these harsh conditions. To that end, extremophile viruses may use a broad variety of tools such as integrases (e.g., SNJ2) [96], which have rapidly gain prominence as genetic tools including, cloning, genome engineering and synthetic biology [97,98,99]. These enzymes can provide an efficient integration of large DNA fragments with different recognition sites and integration mechanisms, which can be utilized to transform microorganisms that are not responsive to standard molecular genetic methods [98].

Reverse transcription (RT) is one of the most important in vitro tools in RNA studies (viral, RNA expression, mRNA biomarkers, non-coding RNAs, etc.), prior further manipulation as copy DNA (cDNA). Currently, RT is done using mesostable enzymes from Moloney murine leukemia virus (MMLV) and avian myeloblastosis virus (AMV). The lack of thermostability of the enzymes limits the detection of certain molecules that have more complicated secondary structures and require higher temperatures for denaturation such as hairpins, stem loops and G quadruplexes, highly structure RNA targets, viral RNA genomes (reviewed in [99]). Screening of thermophilic viral metagenomes has allowed the identification of PyroPhage Pol thermostable enzyme that can be used for both RT and PCR reactions [100,101]. The novel characteristics of the enzyme, along with viral metagenomic information summed to protein engineering resulted in variants of enzymes with novel modified traits [102]. Thus, there is potential in extremophile viral genomes for discovery of new enzymes to be explored and engineered for further applications in molecular biology.

Single-stranded DNA binding (SSB) proteins are ubiquitous across all three domains of life and are found in many viruses, playing essential roles in genome maintenance, DNA replication, recombination, repair and transcription. SSB have been detected in archaeal virus SIRV2. SIRV2 operon containing three genes, gp17, gp18 and gp19 that are highly conserved in rudiviruses and filamentous viruses. gp17 is a SSB protein and differs in structure from the classical SSB, and thus constitutes a novel non-canonical ssDNA binding protein (Guo, Kragelund, White and Peng 2015).

Nucleases have also been found from viromes obtained from acidic hot springs (>85 °C and pH < 3) of Yellowstone National Park. *Acidianus* filamentous virus 1 (AFV1) is an enveloped filamentous virus that infects *Acidianus* species. ORF157 reveals a 157-residue protein that exhibits in vitro nuclease activity that degrades linear dsDNA, and an E86 residue essential for the nuclease activity [103].

DNA polymerases have been also detected in extreme viruses inhabiting circumneutral and alkaline hot springs in Yellowstone National Park and US Great Basin. Bioinformatics and functional screens studies revealed a group of family A-type DNA polymerase (*polA*) genes. The proteins encoded by these viral *polA* genes were remarkably similar to polymerases found in the bacterial phylum Aquificae and the eukaryotic phylum Apicomplexa and invoke a key role of thermophilic viruses in lateral transfer of these polymerase genes which suggests that these genes may be associated with dispersal of diversity-generating mechanisms between geothermal and moderate-temperature biomes [104]. Other polymerases have been detected in extreme metaviromes, particularly from hypoxic estuarine waters obtained in the Gulf of Maine, Dry Tortugas National Park and the Chesapeake Bay (Andrews-Pfannkoch, Fadrosh, Thorpe and Williamson 2010; Schmidt, Sakowski, Williamson, Polson and Wommack 2014).

### 5.2. Nanomedicine and Drug Delivery

At present, there is increasing interest in identifying archaeal viral species with potential for new medical nano-platforms. Viruses such as *Sulfolobus* monocaudavirus 1 (SMV1) and *Sulfolobus* spindle-shaped virus 2 (SSV2) can be efficiently internalized into human cell lines without causing lytic damage; interacting with elements of the innate immune system, and working as activating complementarity [105,106]. Due to their novel biophysical properties, archaeal viruses are becoming increasingly important for research in the fields of bioengineering and nano-therapeutic developments [106]. The discoveries in this regard open new opportunities for studying infection processes, as well as vaccine formulation. Viral-like particles can work as B lymphocyte activators, with additional modification of some of the amino acid residues (lysine, aspartic acid and glutamate) with antigens. A similar approach can also consider to produce cellular biosensor (reviewed in [106]). Furthermore, studying the potato virus X (PVX) has proven beneficial to develop tools used in molecular imaging, tumor homing, drug delivery, vaccination, biosensor design, biomaterials development and biocatalysts [107]. Thus, virus nanoparticles are receiving more and more attention due to their outstanding structural characteristics and ease of functionalization compared to synthetic nanoparticles.

Currently, there is no information that explains the apparent absence of cellular receptors in archaeal viruses, or none has been identified so far. However, there are reports describing the binding of archaeal viruses to extracellular structures such as pili. For instance, *Sulfolobus islandicus* rod-shaped virus 2 (SIRV2), binds to the tips of the pili, with ulterior movement to the cell along the pili. This phenomenon supports the notion that extremophile viruses, and in particular thermophilic viruses, have developed a system to reduce their time outside the cell (reviewed in [21]). This type of movement and cell recognition by the virus virions, has a potential use in virus-like nanoparticles for delivery of molecules of interest in different models. In this process, virions do not contain genetic information (genomic DNA or RNA), instead a molecule of interest (e.g., drug) is carried inside. These virus-like nanoparticles are then delivered and bound specifically to the target cell(s). This approach can be used in increasing the specificity on cancer treatments as the virus-like nanoparticle will only interact with a specific type of cell.

In recent years, in the biopharmaceutical and molecular diagnostics areas, lytic enzymes have gained increasing importance due to their potential use as new strategies against antibiotic-resistant bacterial pathogens in the current age of mounting antibiotic resistance. A number of enzymes have been identified using sequencing-based screens from extremophile viromes, among them, lysin-like genes have been identified from two mildly alkaline hot springs in Yellowstone [5,108]. Endolysins or lysins are phage-encoded enzymes capable of hydrolyzing the bacterial cell wall and are synthesized at the end of the phage replication cycle (Vázquez, García and García, 2018).

For example, from a whole genome study of *Thermus* phage TSP4 isolated from Tengchong hot spring in Yunnan Province of China at a temperature of 70 °C with a pH of 7.0, was identified a novel phage lysin named TSPphg and assessed in vitro and in vivo its antibacterial activity against a panel of antibiotic-resistant strains. TSPphg is able to cause bacteria destruction and has shown bactericidal activity against both Gram-negative and Gram-positive pathogenic bacteria, especially antibiotic-resistant strains of *Klebsiella pneumoniae* [109].

Recently, the first crystal structure of an endolysin (Ts2631) from a thermophilic bacteriophage vB_Tsc2631 isolated from a hot spring of the Hverager geothermal area, Iceland has been reported. The analysis of the structure and sequence of a Ts2631 revealed that it contains a unique N-terminal sequence that is not found in the phage homologs, a 20 residue extension at the N-terminus with a unique motif rich in arginines that is not homologous to any other protein sequence present in the UniProt database and that can protrude from the remainder of the enzyme and is crucial for peptidoglycan binding. Using this unique N-terminal sequence for the design of fusion proteins might become a platform for the development of novel protein antibiotics targeting Gram-negative bacteria [110].

### 5.3. Industrial Biotechnology

Viruses infecting microalgae are responsible for controlling their bloom in the Arctic and Antarctic oceans [74,82]. Due to their ability to control lysis and thus mortality of micro eukaryotes, this property can be explored to produce controlled lysis in industrial cultures of microalgae, for example, for production of biofuels. Viruses in the algae lysis cycle can be controlled via nutrient concentration (e.g., CO_2_, Phosphorus) [111] and activated when the algae production reaches the desired concentration. This approach requires the evaluation of viruses able to lyse commercial species of microalgae and/or their modification (engineering) to the desired effect.

Extracellular polymeric substances (EPS) are produced by different types of microorganisms, in response to environmental stress. As evidenced by different psychrophilic bacteria, EPS production is not only used as cryoprotectant, but also as a defense mechanism against viral attacks [112]. If it is possible to modulate the production of EPS by the presence or absence of viral markers, EPS could be produced in high concentrations and yield, scaling up its production for biotechnological applications with economic feasibility. In this sense, the study of virus–host interactions to enhance EPS expression can be a game changer for EPS industrial and medical applications such as bioplastics, scaffold materials for tissue culture and biomedical polymers [112,113,114].

Another potential application is the use of enzymes able to perform in extreme conditions such as endolysin/lysozyme. Some of those have been described in viruses from extreme environments. These enzymes could be used for treating fouling of filters created by bacterial growth [82]. The potential use of these extremophile enzymes is even more relevant considering that some of these filters are used in environments with extreme pH and salt concentrations [115] which requires the use of enzymes that are fully active at such conditions. It is noteworthy to mention that their effectiveness against microbes have been successfully proven [111]. Phage endolysins have shown a wide range of activity in both high salt environments (100% activity at 500 mM NaCl for *Pseudomonas aeruginosa*-infecting phage fKZ) and high temperatures (activity over 94% at 50 to 78 °C in Ph2119) (reviewed in [112]).

Bacteriophage 9A has several identified enzymatic traits (AMGs) that guarantee activity at low temperature of the host (reviewed in [56]). Thus, the continuous discovery of thermostable enzymes might expand its use for biotechnological processes taking place at extreme temperatures, where specific enzymes functions are desired. Such is the case of studied thermophilic enzymes of great importance to the breakdown of biomass and other materials such as waste plastics [116]. Equally important is the use of psychrophilic enzymes in industrial processes, where instability issues with reactants and products can be avoided by such enzymes, allowing a reduction in costs. This property is due to their lower energy consumption and their high catalytic efficiency [113]. Examples of these are an l-aminoaceylase and a γ-lactamase. Industrial applications of other enzymes such as transaminases, carbonic anhydrases, dehalogenases, esterases and epoxide hydrolases are also being assessed [113].

## 6. Prospectus

Here, we presented an overview of what is known about viruses in extreme environments, highlighting their diversity and the molecular mechanisms that allow them to form complex, symbiotic relationships with a variety of prokaryotic and eukaryotic hosts. Of note, we highlighted the role viruses play in regulating population dynamics in the different ecosystems on earth, mainly those in inhabiting the oceans. We believe that expanding this area of knowledge would result in a more accurate depiction of viral ecology and its biotechnological potential in extreme environments.

**Figure 2:**
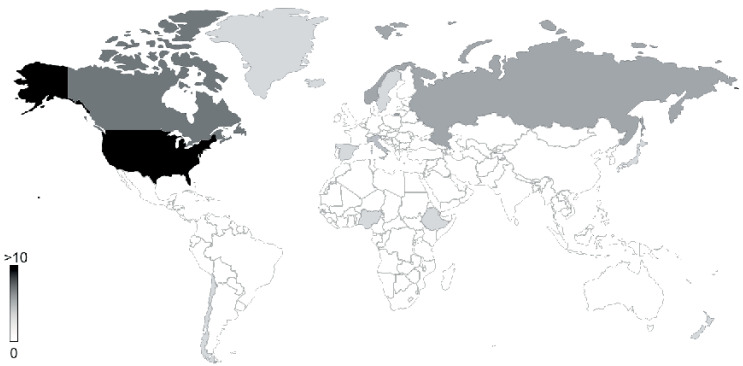
World heat map of virus detected in extreme environments. Locations are colored (gray scale) according to the amount of reviewed literature originating from specific country. Locations are shown by country and represented with a grey scale.

Although new sequencing technologies, as well as sampling methods have facilitated the understanding of the genomics and metabolic mechanisms characterizing viruses in extreme environments, there is still significant knowledge gaps on the dynamics and biological processes of these viruses at a global scale. An example of this limitation is the omission of studies of viruses in the equatorial zone of the world (Africa and Latin America) (Figure 2). For instance, out of 114 reviewed articles on viruses from extreme environments, 109 have been originated from regions in temperate and fridge zones, in fact, USA and Europe contribute to 75% of the reported studied areas. In contrast, despite the presence of 174 volcanic environments in the South American Andes (soil, lakes and hot springs) [117], only 5 reports originate from countries in the equatorial zone, and 0 from continental South America. This unexplored viral diversity provides a unique opportunity to study the extremophile viruses of bacteria, archaea and eukaryotic organisms as novel sources of viruses and viral enzymes with biotechnological potential. Studies in virology in these places can bring new insight about the role viruses have in shaping biodiversity in the equatorial zone, providing an opportunity to develop collaborative research efforts with scientists worldwide to establish a catalogue of extreme viruses and their genomic potential in understudied regions of the world [114,118].

## Figures and Tables

**Table 1:** Main features of the archaeal viruses characterized in extreme environments.

Environment Parameter/ Habitat	Virus (Host)	Family	Capsid Morphology	Additional Features	Genome Type	Isolation Origin	Reference
Temperature >50 °C Hot spring	PFV1 virion (archaeal genus *Pyrobaculum)*	*Tristromaviridae*	Rod-shaped helical nucleocapsid and a nucleocapsid-encompassing protein sheath	Contains an envelope and an inner core	dsDNA	Hyperthermophilic archaea. Pozzuoli Solfatara, Italy	[23]
Hot acidic spring	SSV1 (*Sulfolobus solfataricus* PH1)	*Fuselloviridae*	Icosahedral	six-fold symmetric tail	dsDNA	Hyperthermophilic archaea	[24]
Hot acidic spring	*ATV (Sulfolobus*, *Acidianus*, *Thermoproteus*, *Pyrobaculum)*	*Fuselloviridae*, *Lipothrixviridae*, *Rudiviridae*, *Guttaviridae*, *Globuloviridae*, *Ampullaviridae*	Icosahedral	six-fold symmetric tail	dsDNA	Hyperthermophilic archaea	[25]
Hot springs	Metagenomic search	*Ampullaviridae*, *Bicaudaviridae*, *Lipothrixviridae* and *Rudiviridae*			dsDNA	Metagenomes isolated worldwide	[26]
Hot springs	*Metallosphaera* turreted icosahedral virus (MTIV) (*Metallosphaera yellowstonensis*, MK1)		Icosahedral	Unusual structure that has 42 turret-like projections: 12 from each of the 5-fold axes and 30 hexameric units positioned on icosahedral 2-fold axes	dsDNA	Hot springs water samples collected from Yellowstone National Park	[27]
Hot springs	Thermus virus P23-77 (*Thermus thermophilus*)	*Tectiviridae*	Icosahedral		dsDNA	Alkaline hot spring New Zealand	[28]
Hot springs	SBFV1, SBFV2, SBFV3, SBRV1, SPV1, SPV2, SBV1 (*Sulfolobales*)	*Portogloboviridae*, *Rudiviridae*, *Lipothrixviridae*	Icosahedral	Filamentous and rod-shaped viruses	dsDNA	Hot spring Umi Jigoku in Beppu, Japan	[1]
Temperature 81–96 °C and pH 1–7	*MRV1*, *ARV3*, *SSRV1*, *PSV2*, *PFV2 (Pyrobaculum*, *Saccharolobus*, *Acidianus*, *Metallosphaera)*	*Rudiviridae*, *Globuloviridae* and *Tristromaviridae*		Rod-shaped and spherical viruses	dsDNA	Hyperthermophilic archaea. Pozzuoli Solfatara, Italy	[2]
Temperature 80 °C and pH 3 Saturated salts salinity level (8–36%)	SIRV2 (*Sulfolobus islandicus*)	*Rudiviridae*	Non-enveloped, rod- shaped virus	Flexible filamentous particle 830 nm long and 8 nm wide	dsDNA	Hyperthermophilic acidophilic archaeon *Sulfolobus islandicus*	[29]
Temperature 80 °C and pH 3 Saturated salts salinity level (8–36%)	*HRPV*, *SCTP*, *HRTV*, *HHTV*, *HCTV*, *HHPV*, *HSTV*, *HHIV*, *HJTVHVTV*, *HGTV*, *HATV*, *SSIP*, *HGPV (Halorubrum*, *Haloarcula*, *Halogeometricum*, *Halogranum*, *Salicola*, *Salisaeta)*				ssDNA	Hypersaline water and salt crystal samples collected from Italy, Thailand, Israel; Slovenia and Spain	[30]
Hypersaline environments	*Haloarcula californiae icosahedral virus 1 (HCIV-1)*	*Sphaerolipoviridae*	Icosahedral	Tailed and two non-tailed virus morphotypes	dsDNA	Solar saltern, Thailand	[31]

**Table 2:** Main features of the bacterial viruses characterized in extreme environments.

Environment Parameter/ Habitat	Virus (Host)	Family	Capsid Morphology	Additional Features	Genome Type ^b^	Isolation Origin	Host Features	Reference
Temperature >50 °C Compost pile	GBK2 (*Geobacillus kaustophilus)*	*Siphoviridae*	Icosahedral	Tail (long non-contractile)	dsDNA	Thermophilic bacteria. Cary, NC, USA	*Geobacillus* species are Gram-positive thermophilic bacteria that can ferment C-5 and C-6 sugars to mixed acids and ethanol and have potential for biofuel production	[51]
Temperature 70–90 °C Hot spring sediment	ϕOH3 (*Thermus thermophiles)*	*Inoviridae*	Filamentous	flexible filamentous particle 830 nm long and 8 nm wide	ssDNA	Hyperthermophilic bacterium *Thermus thermophilus* HB8, Obama hot spring, Nagasaki, Japan	Thermusbacteria, with optimal growth temperatures of 70–75 °C, are found in alkaline hot springs, hot water heaters and natural waters subjected to thermal pollution	[48]
Temperature High: 70 °C Alkaline hot springs	115 thermophilic phage strains on seven *Thermus* strains (*T. aquaticus* ATCC 25104, 25105 and 31558, *T. filiformis* ATCC 43280, *T. flavus* ATCC 33923, *T. lacteus* ATCC 31557, *T. rubens* ATCC 31556, *T. ruber* ATCC 35948, *T. thermophiles* ATCC 27634, *Thermus* spp. ATCC 27737, 27978 and 31674)	Myoviridae, *Siphoviridae*, *Tectiviridae* and *Inoviridae*	Icosahedral	*Myoviridae* (phages with contractile tails), *Siphoviridae* (phages with long and noncontractile tails), *Tectiviridae* (isometric capsids) *Inoviridae* (filamentous)	dsDNA, ssDNA	Hyperthermophilic *Thermus* species. Alkaline hot springs in Iceland, New Zealand, Russia (Kamchatka), and the U.S.A.	Thermusbacteria, with optimal growth temperatures of 70–75 °C, are found in alkaline hot springs, hot water heaters and natural waters subjected to thermal pollution	[52]
Temperature Deep-sea hydrothermal fields	*Bacillus* virus W1, BVW1 (*Bacillus* sp. w13)	*Siphoviridae*	Icosahedral	Long tail (300 nm in length and 15 nm in width) and a hexagonal head (70 nm in diameter)	dsDNA	Thermophilic bacteria. east-Pacific and west-Pacific hydrothermal fields		[50]
	*Geobacillus* virus E1, GVE1 (*Geobacillus* sp. E26323)	*Siphoviridae*	Icosahedral	Hexagonal head (130 nm in diameter) and a tail (180 nm in length and 30 nm in width)	dsDNA			
Temperature 0 °C Artic sea ice	1a *(Shewanella frigidimarina)*, 11b *(Flavobacterium hibernum)*, 21c *(Colwellia psychrerythraea)*	*Myoviridae/Siphoviridae*	Icosahedral	Tailed	dsDNA	Marine psychrophilic phage-host systems from Svalbard (Arctic)		[55]
Temperature −12 and 8 °C	9A (*Colwellia psychrerythraea* 34H)	*Siphoviridae*	Icosahedral	Long and flexible tail	dsDNA	Marine psychrophilic	*Colwellia psychrerythraea* are gammaproteobacteria isolated fromArctic marine sediments), is a model psychrophile that grows at temperatures from −12 to 19 °C with optimal growth at 8 °C.	[56]
pH Low: 0–2 °C Acid mine drainage	AcaML1 (*Acidithiobacillus caldus* ATCC 51756)	*Myoviridae*	Icosahedral	Tail (contractile)	dsDNA	Acidophile bacteria, Chile	*Acidithiobacillus* are gammaproteobacteria that are ubiquitous in biomining biotopes, with several characterised species that play key roles in industrial metal recovery	[54]
Saturated salts 7.5% NaCl (wt/vol) Hypersaline soils	F9-11 (*Deleya halophile*)	-	-	Isometric head and non-contractile tail	-	Halophilic bacteria Alicante (Spain)	*Deleya halophile* F9-11	[57]
Saturated salts Hypersaline soils	ΦgspA, ΦgspB, ΦgspC, ΦgspD and ΦgspE (*Halomonas* spp.)	*Myoviridae*	Icosahedral	Tailed	dsDNA	Great Salt Plains National Wildlife Refuge (GSP). North-central Oklahoma, USA	*Halomonas* spp.	[58]
Saturated salts 6% NaCl (wt/vol) Soda lakes	Mgbh1, Shbh1 (*Bacillus* sp.) Shpa (*Paracoccus marinus*)	*Myoviridae* *Siphoviridae*	Icosahedral	Tailed	dsDNA	East African Rift Valley soda lakes	*Bacillus* sp./*Paracoccus marinus. Bacillus- and Paracoccus* species have important roles in biogeochemical cycling in soda lakes	[59]
High hydrostatic pressure (~381 mbsf) ^a^ Deep subsea floor sediments	RR1-A RR1-B (*Rhizobium radiobacter*)	-	-	-	-	Peru margin area to the open ocean of the eastern equatorial Pacific	*Rhizobium radiobacter* is the most frequently isolated and highly abundant representative of the marine deep subsurface	[60]

^a^ mbsf (meters below seafloor); ^b^ Single-stranded DNA (ssDNA), double-stranded DNA (dsDNA).

**Table 3:** Virus in extremophile animals.

Virus Type	Known Host	Isolation Origin	Geographic Range	Genes or Molecular Traits Followed	Genome Size and Other Genetic Traits	Accession Numbers	References
Eukaryotic circular Rep‒ncoding ssDNA (CRESS‒DNA) viruses	Marine invertebrates	Deep sea	‒	Metagenomics analysis Putative cap	Eukaryotic circular Rep-encoding ssDNA	KR528543 to KR528569	[66]
Eukaryotic virus SSDNA	‒	Deep sea hadopelagic sediments	Pacific Ocean japan	Methagenomics analysis genetic markers (major capsid protein [VP1] and replication protein [Rep])	Eukaryotic virus ssDNA	DRA000564 BAKA01000001 to BAKA01000006 BAKB01000001 to BAKB01000011 BAKC01000001 to BAKC01000114	[67]
Phocine herpesvirus 1 (PhHV‒1)	Harbor seals (Phoca vitulina)	Polar	Norway	glycoprotein B gene	Large dsDNA	‒	[68]
Kigluaik phantom virus (KIGV) Nome phantom virus (NOMV)	Phantom midges (*Chaoborus*)	Polar	Arctic	The endonuclease domain to motif E of the conserved polymerase domains	3 (‒) ssRNA segments	KJ434182 to KJ434187 KJ461793 to KJ461811	[65]

**Table 4:** Virus in extremophile eukaryotic microorganism.

Virus Type	Known Host	Isolation Origin	Geographic Range	Genes or Molecular Traits Followed	Genome Size and Other Genetic Traits	Accession Numbers	References
Eukaryotic viruses	Algae amoeba	Polar	Arctic	Metagenomics analysis	dsDNA	‒	[72]
Micromonas pusilla viruses (MpV)	Micromonas pusilla	Low phosphorous and polar	Arctic	DNApo)	dsDNA	‒	[69]
*Micromonas pusilla* viruses (MpV) MpV‒08T	Micromonas pusilla	Low phosphorous and polar	Arctic	‒	dsDNA	‒	[74]
P. globose viruses (PgV) PgV‒07T	P. globosa	Low phosphorous and polar	Arctic	‒	dsDNA	‒	[74]
Micromonas pusilla viruses (MpV) MpoV-44T, 45T, 46T and 47T	Micromonas pusilla	Polar	Arctic	DNA polymerase B gene (polB)	dsDNA 205 kbp, 191 kbp, 192 kb, 190 kbp	ky682859 to ky682862	[82]
*Micromonas pusilla* viruses (MpV) MpV‒08T	Micromonas pusilla	Polar	Arctic	Viral major capsid protein	dsDNA	‒	[73]
Emiliania huxleyi specific viruses (EhVs)	Emiliania huxleyi	Polar	North Sea	MCP gene	dsDNA	DQ084403 to DQ084406	[83]
EhV‒99B1 and EhV86	Emiliania huxleyi	Polar	Norwegian fjord	EhV‒86 phosphate permease, endonuclease CDS, putative transposase CDS, tRNAs	dsDNA 410 kbp, 160–180 nm	FN429076	[75]
EhV	Emiliania huxleyi	Polar	Norwegian fjord	‒	dsDNA	‒	[84]
E. huxleyi lytic virus EhV201 Phycodnaviridae	Emiliania huxleyi	Polar	Norwegian fjord	Sphingolipid metabolism	‒	‒	[70]
Cafeteria roenbergensis virus (CroV),	C. roenbergensis strain	Deep sea		Genome sequencing DNA polymerase	dsDNA 730 kb	GU244497	[85]
Coccolithoviruses	Emiliania huxleyi	Deep sea	Norway	Metagenomic analysis Genes ehv452 and ehv060	dsDNA virus monopartite >350 kbp	PRJEB5540	[77]
Curvularia thermal tolerance virus	Curvularia protuberata	Geothermal soil		qrtpcr	dsRNA	70403454 to 70407660.	[79]
4 Yellowstone Lake virophages,	Algae	Geothermal	Yellowstone Lake	Metagenomics MCP, DNA polymerase B family, poxvirus late transcription factor, topoisomerase II, vaccinia virus, A32‒like packaging ATPase, ribonucleotide reductase small subunit, multidrug resistance protein, OLV ORF2	dsDNA 178 kbp, 171 kbp, 17 kbp, 73 kbp	LC015646‒LC015649	[71]
Nucleocytoplasmic large DNA viruses (NCLDV)	Different microorganisms	Different environments	In silico	Bioinformatics analysis ATPases, NCLDV ATPases and polinton ATPases	dsDNA 100 kbp to 2.50 Mbp	-	[86]
Yellowstone Lake virophages —YSLV5, YSLV6 and YSLV7	Giant DNA viruses and eukaryotic hosts	Geothermal	Yellowstone Lake	Metagenomics ATPase, MCP and Pro	cdsDNA 30 kbp, 25 kbp, 24 kbp	KM502589 to KM502591	[87]
Organic Lake virophage (OLV)	Acanthamoeba polyphaga Acanthamoeba polyphaga mimivirus (APMV)	Polar hypersaline meromictic lake	Antarctica Organic Lake	Metagenomics MCP	cdsDNA 18 kbp	HQ704801 to HQ704808	[88]

**Table 5:** Virus in extremophile plants.

Virus Type	Known Host	Extreme Environment	Geographic Range	Genes or Molecular Traits Followed	Genome Size and Other Genetic Traits	Accession Numbers	References
Ancient caribou feces associated virus (aCFV)	‒	Polar	Arctic environment	Rep proteins	small circular ssDNA 2.2 kb	KJ938716	[81]
Ancient Northwest Territories cripavirus (aNCV)	‒	Polar	Arctic environment	RNA‒dependent RNA polymerase	ssRNA 1.8 kb	KJ938718	[81]
Turnip mosaic virus (TuMV)	Arabidopsis (Arabidopsis thaliana)	Drought	‒	Transcriptome and metabolome	(+)ssRNA	GSE46760	[89]
Cauliflower mosaic virus (CaMV)	Turnip (*Brassica rapa* cv. ‘Just Right’)	Drought	‒	Ca4443	dsDNA	‒	[90]
Turnip mosaic virus (TuMV)	Turnip (*Brassica rapa* cv. ‘Just Right’)	Drought	‒	Tu8907	(+)ssRNA	‒	[90]
